# Retraction: Empowering leadership: A conflict resolver and a performance booster for organizations

**DOI:** 10.1371/journal.pone.0362642

**Published:** 2026-11-12

**Authors:** The PLOS One Editors

The *PLOS One* Editors retract this article [1] due to concerns about potential manipulation of the publication process. These concerns call into question the validity and provenance of the reported results.

Additionally, the article cited as Reference 49 was retracted before [1] was published, and the articles cited as References 6 and 53 were retracted after [1] was published.

We regret that the issues were not identified prior to the article’s publication.

The author either did not respond directly or could not be reached.
